# Using the Natural Experiment Study Design to Evaluate the Effect of a Change in Doctor’s Roster on Patient Flow in an Emergency Department

**DOI:** 10.5811/westjem.2018.5.36750

**Published:** 2018-06-08

**Authors:** Peter Hallas, Dan Brun Pedersen

**Affiliations:** Zealand University Hospital, Department of Emergency Medicine, Lykkebækvej, Køge, Denmark

## Abstract

**Introduction:**

The effect of changes in doctors’ rosters is rarely subjected to scientific evaluation. We describe how a natural experiment (NE) study design can be used to evaluate if a managerial decision about doctors’ rosters has an effect on patient flow in an emergency department (ED). We hypothesized that an extra doctor each morning from 6 a.m. (i.e., a modified “casino shift”) might improve the productivity of a hospital’s ED.

**Methods:**

This was an NE observational study using data on patient flow in the ED of Zealand University Hospital, Denmark, between April 1, 2016, and April 1, 2017. We compared days on which the 6 a.m. emergency physician called in sick (case days) with data from the same weekday a week later where staffing was as scheduled (control days).

**Results:**

Patient caseload did not did differ significantly on days with and without the extra doctor from 6 a.m. (measured by number of admissions, triage scores and mean patient age). Door-to-doctor time was 70 minutes (mean, standard deviation [SD], 49) on days without the extra doctor and 56 minutes (mean, SD 41) on days with the early-morning doctor present (p > 0.05). ED length of stay was 250 minutes (mean, SD 119) on days without the extra doctor and 209 minutes (mean, SD 109) on days with the early-morning doctor present (p > 0.05).

**Conclusion:**

In our setting, an extra doctor in the ED from 6 a.m. did not change patient flow. These results suggest that the workflow in the ED should be viewed as a connected supply chain. The study also demonstrates that a natural experiment study design can be used to evaluate ED managerial decisions.

## INTRODUCTION

It is rare that managerial decisions in healthcare about staff rosters, for example, are evaluated using the same scientific approach as would otherwise be required in healthcare interventions^1^ This is surprising given the influence that the rosters can have on the wellbeing of the staff and possibly even the patient flow. Staff fatigue on night shifts is a common problem in emergency departments (ED),^2^ and during shifts resident productivity falls.^3^,^4^

A so-called “casino shift” where handover takes place during the early hours of the morning (e.g., at 4 a.m.) has been suggested as a way of reducing physician fatigue compared to a shift where the handover takes place at the beginning of normal office hours.^5^,^6^ At the Department of Emergency Medicine at Zealand University Hospital, Denmark, we introduced a modified “casino shift” in the spring of 2016. We hypothesized that an extra doctor each morning from 6 a.m. might improve productivity in the ED. To investigate this hypothesis we used a natural experiment (NE) study design. The NE approach is a type of observational study used for evaluation of situations where a controlled experiment is difficult, if not impossible, to conduct. A situation can be analyzed as an NE if outside factors introduce an element of randomness.^7^

## METHODS

The “early morning shift” in our ED was introduced to comply with new labor agreements for junior doctors. The new agreements require that doctors rest an average of 2–3 hours if a shift exceeds 13 hours. This “early morning shift” starts at 6 a.m. on weekdays. The doctor on the “early morning shift” will join the evening/night team of four residents (three in their first year and one in second year or beyond with a consultant on call); the evening/night team is on call from 4 p.m.–8 a.m. Doctors who arrive at 6 a.m. will typically relieve the most experienced doctor on call. The early morning shift was introduced in our ED on April 1, 2016.

If the early-morning doctor calls in sick the vacant shift will not be filled, leaving the on-call evening/night team to handle all patients. Using these “sick-days” as the random element in our study set-up, we compared data from days where the 6 a.m. doctor called in sick (case days) with data from the same weekday a week later (where staffing was as scheduled [control days]). If a control day fell on a public holiday or on another day with a 6 a.m. doctor who called in sick, the weekday in the preceding week was used as control.

In compliance with the regulations on the use of administrative data in the Danish Health Act,^8^ we retrieved data on patient flow in our ED from the electronic flow management system (IMATIS^R^ Fundamentum Platform) from April 1, 2016, to April 1, 2017. Workflow data was included if the patient arrived at the ED between 5 a.m. and 8 a.m. The triage system used a scale from 1 – 5 to signal urgency, with category 1 being the most urgent. We defined door-to-doctor time as the interval between a patient’s arrival and the first registration in the allocated doctor’s flow management system; thus, the triage process was not included, even though all patients are seen by a doctor at the triage station shortly after arrival. Admission to the observational unit was considered as “departure from the ED” even though it remains part of the ED’s area of responsibility. Some patient categories are routinely treated by doctors/teams from other departments of the hospital with little involvement of our emergency physicians. These categories (major trauma; out-of-hospital cardiac arrest; patients with ear/nose/throat-related problems) were not included in the material and neither were patients with minor injuries who were not admitted.

Statistical analysis used t-test and chi-square test with a significance level of 0.05. We analyzed the following data: time of arrival; departure from the ED; triage score; patient age; door-to-doctor time; and next destination for the patient (admission to or discharge from hospital).

## RESULTS

During the 52 weeks of our study there were 16 case-days with a total of 37 patient visits. On the 16 controls days, there were a total of 26 visits (p>0.05). Data on triage were not available for four patients (control days) and time from door to doctor was not available for one patient (control day). On case-days, 48% (n=18) of patients had a length of stay (LOS) of more than four hours, while this was the case in 38 % (n=10) on control days (p>0.05). Days with and without the 6 a.m. doctor did not differ significantly for patient caseload (measured as number of admissions, triage score, or patient age). Door-to-doctor time was 70 minutes (mean, standard deviation [SD], 49) on case-days and 56 minutes (mean, SD 41) on control-days (p > 0.05). ED LOS was 250 minutes (mean, SD 119) on days without the extra doctor and 209 minutes (mean, SD 109) on days with the early-morning doctor present (p > 0.05).

## DISCUSSION

Our study shows that a doctors’ roster incorporating a modified “casino shift” reduces neither the door-to-doctor time nor LOS for patients admitted between 5 a.m. and 8 a.m. in our ED. The results illustrate that the concept of NEs can be used to evaluate managerial decisions in the ED without setting up a costly and time-consuming traditional, randomized controlled experiment. Although the NE research design is well known in fields such as economics, it is not used much in healthcare research. Rockers et al. argue that the NE may have “unrealized potential for … causal evaluation of health policies and programs globally.”^9^ Thus, this design could be considered a complementary approach to gaining insights into the effectiveness of healthcare initiatives.

That rationale for introducing a casino-style, early-morning shift was to relieve resident fatigue, rather than to increase productivity. However, since resident productivity falls during shifts^3^,^4^ it would be a likely “side effect” of a casino shift that productivity on a department level didn’t fall. So why doesn’t adding an extra, rested doctor from 6 a.m. on weekdays in the ED have any significant effect on patient flow? The described “dip” in productivity at the end of shifts described by others^4^ might not be applicable to doctors working in Danish hospitals under Danish labor agreements, partly because the labor agreements mandate that on-call doctors should have opportunities to rest. This might explain why there was no decrease in door-to-doctor time when a vigorous colleague arrived to help out at 6 a.m.

Another explanation could be that other factors than just the number of doctors at work determine the rate of workflow in the ED. This explanation is supported by the fact that LOS in the ED was unchanged between the groups. It is well described in the literature that the rate of patient flow in the ED is determined by multiple factors, not just number of staff.^10^ Other factors could limit the rate of patient flow and the rate with which doctors can see and treat patients in the ED, for example, the sequence of clinical working processes or availability of radiology services and lab tests. Thus, the results we present here point toward the idea that the workflow in the ED should be viewed as a connected supply chain and that no single intervention will improve workflow unless rate-limiting processes are identified first.

Although this study could not document any effect on LOS and door-to-doctor time, there may be other, secondary impacts of the early-morning shift that were not evaluated in this study, e.g., impacts later in the day. Indeed, the labor agreement and the shift reflect attempts to reduce fatigue, rather than increase productivity. More importantly, using LOS and door-to-doctor time as metrics does not indicate whether there was an effect on the quality of care.

## LIMITATIONS

Our study has the inherent limitations of an observational study. In addition, the relatively few number of days with sick staff resulted in a small sample size. A small sample size made it difficult to eliminate other confounding variables. However, a power calculation (using the current incidence of sick days and a standard deviation of 110 minutes) shows that it would require management data from seven years of ED operations to evaluate whether the change in roster increased patient flow. Even if this were done, the absolute risk reduction for a LOS > 4 hours would be 10% (48 minus 38), with a number needed to treat of 10 patients. Thus, in our setting, this equals three to four mornings with a casino shift to reduce door-to-doctor time with 14 minutes. Evaluation of a minor change in a roster by using a study period of seven years to prove a very small effect for a small group of patients would hardly be justifiable. Another limitation is that the results in this study might not be generalizable to other settings where the caseload, staffing or labor agreements differ significantly from our ED.

## CONCLUSION

In our departmental setting, a modified “casino shift” did not change patient flow; however, the results of our study illustrate that the concept of natural experiments can be used to evaluate managerial decisions in the ED.
